# Incident cardiac arrhythmias associated with metabolic dysfunction-associated steatotic liver disease: a nationwide histology cohort study

**DOI:** 10.1186/s12933-023-02070-5

**Published:** 2023-12-13

**Authors:** Tracey G. Simon, Fahim Ebrahimi, Bjorn Roelstraete, Hannes Hagström, Johan Sundström, Jonas F. Ludvigsson

**Affiliations:** 1 Division of Gastroenterology and Hepatology, Massachusetts General Hospital, Boston, MA USA; 2 Harvard Medical School, Boston, MA USA; 3 Clinical and Translational Epidemiology Unit, Massachusetts General Hospital, Boston, MA USA; 4 Department of Medical Epidemiology and Biostatistics, Karolinska Institutet, Stockholm, Sweden; 5 Department of Gastroenterology and Hepatology, University Digestive Health Care Center Basel–Clarunis, Basel, Switzerland; 6 Division of Hepatology, Department of Upper GI Diseases, Karolinska University Hospital, Stockholm, Sweden; 7 Department of Medicine, Karolinska Institutet, HuddingeStockholm, Sweden; 8 Department of Medical Sciences, Uppsala University, Uppsala, Sweden; 9 The George Institute for Global Health, University of New South Wales, Sydney, Australia; 10 Department of Family Medicine and Community Health, Department of Population Health Science and Policy, Icahn School of Medicine at Mount Sinai, New York, NY USA; 11 Department of Pediatrics, Orebro University Hospital, Örebro, Sweden; 12 Department of Medicine, Columbia University College of Physicians and Surgeons, New York, NY USA

**Keywords:** MASLD, Epidemiology, Fibrosis, Arrhythmia, Cardiovascular

## Abstract

**Background:**

Prior studies suggest a link between metabolic dysfunction-associated steatotic liver disease (MASLD) and incident arrhythmias, including atrial fibrillation (AF). However, robust data are lacking from cohorts with liver histology, which remains the gold standard for staging MASLD severity.

**Methods:**

This population-based cohort included all Swedish adults with histologically-confirmed MASLD and without prior cardiac arrhythmias (1966–2016; n = 11,206). MASLD was defined from prospectively-recorded histopathology, and characterized as simple steatosis, non-fibrotic steatohepatitis (MASH), non-cirrhotic fibrosis, or cirrhosis. MASLD patients were matched to ≤ 5 controls without MASLD or arrhythmias, by age, sex, calendar year and county (n = 51,856). Using Cox proportional hazards modeling, we calculated multivariable-adjusted hazard ratios (aHRs) for incident arrhythmias (including AF, bradyarrhythmias, other supraventricular arrhythmias, ventricular arrhythmias/cardiac arrest).

**Results:**

Over a median follow-up of 10.8 years, incident arrhythmias were confirmed in 1351 MASLD patients (10.3/1000 person-years [PY]) and 6493 controls (8.7/1000PY; difference = 1.7/1000PY; aHR = 1.30, 95%CI 1.22–1.38), and MASLD patients had significantly higher rates of incident AF (difference = 0.9/1000PY; aHR = 1.26, 95%CI 1.18–1.35). Rates of both overall arrhythmias and AF were significantly elevated across all MASLD histological groups, particularly cirrhosis (differences, 8.5/1000PY and 5.3/1000PY, respectively). In secondary analyses, MASLD patients also had significantly higher rates of incident ventricular arrhythmias/cardiac arrest (aHR = 1.53, 95%CI 1.30–1.80), bradyarrhythmias (aHR = 1.26, 95%CI 1.06–1.48), and other supraventricular arrhythmias (aHR = 1.27, 95%CI 1.00–1.62), compared to controls.

**Conclusions:**

Compared to matched controls, patients with biopsy-confirmed MASLD had modest but significantly higher incidence of cardiac arrhythmias, including AF, bradyarrhythmias, other supraventricular arrhythmias and ventricular arrhythmias/cardiac arrest. Excess risk was observed across all stages of MASLD and was highest with cirrhosis.

**Supplementary Information:**

The online version contains supplementary material available at 10.1186/s12933-023-02070-5.

## Introduction

Metabolic dysfunction-associated steatotic liver disease (MASLD) represents a leading cause of chronic liver disease in the United States (U.S.) and Europe, where it affects over 100 million adults [1, 2]. Between 20 and 33% of patients with MASLD develop progressive steatohepatitis (MASH) with fibrosis, which in turn may progress to cirrhosis, decompensated liver disease, and liver-related mortality [3–7]. Observational data have also linked MASLD to the development of diverse cardiovascular complications, including coronary atherosclerosis [8, 9], cardiac diastolic dysfunction [10], and both subclinical [8], and overt cardiovascular (CV) events [11, 12]. Based on this body of evidence, both the American Association for the Study of Liver Disease (AASLD) and the European Association for the Study of the Liver (EASL) recommend that patients with MASLD undergo careful cardiovascular screening [13, 14].

Recent data further suggest that MASLD may adversely impact cardiac conduction and autonomic function, and could contribute to the development of cardiac arrhythmias [15, 16], including atrial fibrillation [17, 18], the most common arrhythmia and a cause of significant morbidity and mortality [19]. However, published data are limited and conflicting, with some observational studies reporting increased rates of atrial fibrillation and other arrhythmias in MASLD [20, 21], while others have found conflicting results [17, 22]. Among the few prior studies in this area, all have had substantial limitations, including a focus on prevalent – rather than incident – arrhythmias [23–25], or reliance upon surrogate, non-invasive definitions of MASLD [17, 20–22], which are prone to misclassification, and cannot identify MASH or fibrosis severity. To date, only one longitudinal study has included liver histology [24], but it derived from a single center and had a small sample size (N = 215), with very few events and limited generalizability.

Thus, we leveraged a nationwide histopathology cohort, comprised of all adults in Sweden with biopsy-confirmed MASLD, to examine the incidence of cardiac arrhythmias according to the presence and histological severity of MASLD.

## Methods

### Study population and MASLD ascertainment

Within the ESPRESSO (Epidemiology Strengthened by Histopathology Reports in Sweden) cohort, we conducted a population-based, matched cohort study. ESPRESSO encompasses prospectively-recorded liver histopathology submitted to all 28 pathology departments in Sweden between 1965 and 2016 [26]. Each liver biopsy report includes liver biopsy date and location, the unique personal identity number (PIN) assigned to all Swedish residents, and describes topography within the liver and morphology, using the Systematized Nomenclature of Medicine (SNOMED) system. We linked ESPRESSO to validated, nationwide registers that include comprehensive data regarding patient demographics, clinical comorbidities, prescription medications, incident cardiac arrhythmias and death. Informed consent was waived as the study was register-based [27].

For the current study, we identified all adults aged ≥ 18 years with a liver biopsy between 1966–2016 that confirmed MASLD, as defined as at least one morphology code for steatosis (M5008x or M5520x) without another etiology of liver disease or cardiac arrhythmia, on or before the index date (i.e. date of liver biopsy), using a validated international classification of disease (ICD) algorithm (Additional file 1: Figure S1; eMethods) [12]. As in prior studies, we excluded anyone with another recorded etiology of liver disease, alcohol abuse/misuse, liver transplantation, cardiac arrhythmia or emigration from Sweden, on or before the index date (Additional file 1: Table S1).

In a previous validation study [12], this methodology yielded a positive predictive value (PPV) of 92% for MASLD overall. All patients who met our criteria for MASLD were further classified at the time of their index biopsy into one of four mutually exclusive categories of histological severity (i.e. simple steatosis, MASH, without fibrosis, non-cirrhotic fibrosis and cirrhosis). Specifically, simple steatosis was defined by at least 1 code for steatosis and no additional codes for inflammation (i.e. M5400x or M4-) or fibrosis (i.e. M4900x) or cirrhosis (i.e. M4950x). MASH without fibrosis was defined broadly by the presence of at least 1 code for steatosis plus at least 1 code for inflammation (i.e. M5400x or M4-), without any codes for fibrosis or cirrhosis. Non-cirrhotic fibrosis (i.e. F1-F3 fibrosis, with or without MASH) was defined by the presence of at least 1 code for steatosis plus at least 1 code for fibrosis (i.e. M4900x), but no codes for cirrhosis. Cirrhosis was defined by at least 1 code for cirrhosis (i.e. M4950x). This algorithm has been validated and yielded high positive predictive values (PPV) for each subcategory: simple steatosis (PPV 90%), MASH without fibrosis (PPV 87%), non-cirrhotic fibrosis (PPV 93%), and cirrhosis (PPV 97%) [12]. Each MASLD patient was then subsequently matched to up to five general population controls without recorded MASLD or prior cardiac arrhythmia, according to age, sex, calendar year and county of residence. Population controls were derived from the Total Population Register [28], with identical exclusion criteria (Additional file 1: Figure S1).

### Outcomes and covariates

Outcomes and covariates were ascertained from the validated Patient Register, which prospectively records data from all inpatient and outpatient medical facilities in Sweden, including hospital discharge diagnoses (since 1964) and specialty outpatient care (since 2001), with established PPVs for clinical diagnoses between 85 and 95% for liver-related and cardiovascular diseases [29], including PPV of 97% for atrial fibrillation in prior validation studies [30]. The two primary outcomes were: (1) incident overall arrhythmias (a composite defined by ≥ 1 primary or secondary inpatient or outpatient ICD diagnosis for atrial fibrillation or atrial flutter, bradyarrhythmia, other supraventricular arrhythmia, ventricular arrhythmias or cardiac arrest), and (2) incident atrial fibrillation (definitions in Additional file 1: Table S2). In prior validations, such ICD-based algorithms for identifying arrhythmias yield PPVs > 84% [31–35]. Secondary outcomes included other, individual arrhythmias (bradyarrhythmias, other supraventricular arrhythmias, and ventricular arrhythmias/cardiac arrest). In sensitivity analyses we also applied a more stringent definition of arrythmias (requiring ≥ 2 inpatient or outpatient diagnoses separated by > 6 months, with outcome diagnosis date defined by the last diagnosis). Mortality was ascertained from the Total Population Register, which records 93% of all deaths in Sweden within 10 days, and the remaining 7% within 30 days.

Detailed information was collected regarding demographics, education, comorbidities and prescription medication use (Additional file 1: Table S3; eMethods). Briefly, age at the index date (i.e. biopsy date among MASLD patients, or corresponding matching date among controls), sex, date of birth and emigration status were ascertained from the Total Population Register [28], and education level was obtained from the Longitudinal integrated database for health insurance and labour market studies. Data on education were missing for 3.1% of adults with an index date on or after January 1, 1990, which was the first year in which the LISA database was introduced [36]. We included a separate missing category for missingness on education level but did not impute any missing data.

Comorbidities were extracted from the Patient Register using established ICD algorithms [29] (Additional file 1: Table S3; eMethods), and we also identified the number of hospitalizations for each patient in the year preceding the index date. The Total Population Register also includes data from the Multigenerational Register regarding first-degree family members, which permitted us to ascertain a first-degree family history of early cardiovascular disease (CVD) diagnosed before age 50 years. Additionally, we collected detailed data regarding use of the following medications: statins, other lipid-lowering agents, low-dose aspirin (< 163 mg), other anti-platelets, anticoagulants, antidiabetic medications and anti-hypertensive agents [37]. Medication use was ascertained from the Prescribed Drug Register, a well-validated and virtually-complete nationwide register [37], that includes prospectively-recorded data for all dispensed prescriptions from Swedish pharmacies since July, 2005.

### Statistical analysis

Our primary analyses examined rates of incident arrhythmias according to the presence and histological severity of MASLD, compared to matched controls. Follow-up began at the index date (or matching date for controls), and continued to the first recorded study outcome, death, emigration, or end of follow-up (December 31, 2016). For all outcomes, we calculated incidence rates and absolute rate differences, together with 95% confidence intervals (CIs). Using cause-specific Cox proportional hazard regression models, we estimated multivariable adjusted hazard ratios (aHRs) and 95%CIs for incident arrhythmias, and defined death due to non-cardiac causes as a potential competing event. The fully-adjusted multivariable model accounted for matching factors (i.e. age, sex, calendar year and county) and a priori-selected confounders defined up to and including the index date (i.e. diabetes, obesity, hypertension, dyslipidemia, chronic kidney disease, a first-degree family history of early CVD before age 50 years, education, number of inpatient hospitalizations in the year before the index date, and finally diagnoses of alcohol abuse/misuse recorded during study follow-up [12]) (eMethods; Additional file 1: Table S3). We did not adjust for heart failure or coronary artery disease since they constitute intermediates but are unlikely to be true confounders [38]. The proportional hazards assumption was assessed by examining the relationship between Schoenfeld residuals and time.

To better characterize the gradient of risk associated with MASLD histological severity, and to minimize potential confounding from misclassification of non-MASLD controls, we restricted the cohort to patients with MASLD, with simple steatosis as the comparator [12]. In stratified analyses, we examined the associations between MASLD and incident arrhythmias according to sex, and categories of age, follow-up duration, and calendar year of index biopsy, and we tested the significance of effect modification between J groups using the contrast test statistic, which approximates a chi-squared distribution with J-1 degrees of freedom under the null. [39]

To assess potential confounding related to shared genetic or early environmental factors, including a first-degree family history of early CVD, we identified all MASLD patients with ≥ 1 full sibling without recorded MASLD or arrhythmia before the start of follow-up, and otherwise applied identical exclusion criteria. We then compared each MASLD patient with his or her full sibling(s), after conditioning on matching set within family, and further adjusting for all covariates in the multivariable model.

We conducted several sensitivity analyses to test the robustness of our results. First, because a widely-used histological scoring system was published in 2005 [40], the same year that comprehensive prescription medication data was first available in Sweden, we restricted the cohort to patients with index date ≥ January 1, 2006, and constructed models further accounting for relevant medication use (i.e. aspirin, other antiplatelets, statins, other lipid-lowering agents, antidiabetic agents and/or anti-hypertensive medications). Second, because we lacked detailed data regarding smoking, we also constructed models further adjusting for chronic obstructive pulmonary disease (COPD), as a proxy for heavy smoking. Third, we computed an “E-value” [41], which is the minimum strength of the association that an unmeasured confounder would need to have with both the exposure and the outcome, to fully attenuate the observed association between MASLD and the study outcome. Fourth, because a diagnosis of underlying CVD might mediate an association between MASLD and incident cardiac arrhythmias, we constructed separate multivariable models focused on primary arrhythmias, by excluding patients with prior, underlying CVD, and further censoring patients during follow-up at the time of a CVD diagnosis. Finally, to address potential reverse causation, we repeated our analysis after excluding patients with incident arrhythmias recorded within < 90 days or within < 2 years of follow-up (with follow-up therefore starting at day 90 or at 2 years, respectively).

Analyses were conducted using R software (version 3.6.1, R Foundation for Statistical Computing, Vienna, Austria; survival package version 2.44 [Therneau, 2015, https://CRAN.R-project.org/package=survival]). A two-sided P < 0.05 was considered statistically significant.

### Role of the funding source

No funding organization had any role in the design and conduct of the study; in the collection, management, and analysis of the data; or in the preparation, review, and approval of the manuscript.

## Results

Table 1 outlines the baseline characteristics of 11,206 adults with histologically-confirmed MASLD and 51,856 matched general population controls. Among MASLD patients, the average age at index biopsy was 53 years, and 45% were female. Simple steatosis was found in 7,642 (68.2%), while 1,257 (11.2%) had MASH without fibrosis, 1695 (15.1%) had non-cirrhotic fibrosis, and 612 (5.5%) had cirrhosis. MASLD patients were more likely than controls to have diabetes, obesity, hypertension, dyslipidemia and chronic kidney disease.

**Table 1:** Baseline Characteristics of Adults with Histologically-Confirmed MASLD and Matched Population Controls, at the Index Date (1966–2016)*

Characteristic	Population controls N = 51,856	All MASLD N = 11,206	Simple Steatosis N = 7642	MASH without fibrosis N = 1257	Non-Cirrhotic Fibrosis N = 1695	Cirrhosis N = 612
Sex, n (%)						
Men	28,055 [54.1]	6140 [54.8]	4236 [55.4]	652 [51.9]	913 [53.9]	339 [55.4]
Women	23,801 [45.9]	5066 [45.2]	3406 [44.6]	605 [48.1]	782 [46.1]	273 [44.6]
Age						
Mean (SD)	52.68 [14.7]	53.2 [14.8]	52.3 [15.0]	52.9 [15.2]	55.1 [14.1]	59.4 [11.6]
Median (IQR)	54.0 [42.0–64.0]	55.0 [43.0–64.0]	53.0 [41.0–64.0]	54.0 [42.0–64.0]	57.0 [46.0–66.0]	62.0 [53.0–67.0]
Country of birth, n (%)						
Nordic country	47,406 [91.42]	10,070 [89.86]	6927 [90.64]	1113 [88.54]	1477 [87.14]	553 [90.36]
Other	4447 [8.58]	1136 [10.14]	715 [9.36]	144 [11.46]	218 [12.86]	59 [9.64]
Missing	3 [0.01]	0 [0.00]	0 [0.00]	0 [0.00]	0 [0.00]	0 [0.00]
Education level^1^, n (%)						
≤ 9 years	2986 [22.08]	682 [22.19]	342 [20.90]	96 [22.91]	197 [23.65]	47 [25.27]
10–12 years	5902 [43.65]	1406 [45.74]	727 [44.44]	214 [51.07]	376 [45.14]	89 [47.85]
≥ 13 years	4364 [32.27]	740 [24.07]	419 [25.61]	76 [18.14]	214 [25.69]	31 [16.67]
Missing	270 [2.00]	246 [8.00]	148 [9.05]	33 [7.88]	46 [5.52]	19 [10.22]
Start of follow-up, year						
1966–1989	9164 [17.67]	1906 [17.01]	1535 [20.09]	145 [11.54]	114 [6.73]	112 [18.30]
1990–2005	30,412 [58.65]	6501 [58.01]	4632 [60.61]	725 [57.68]	816 [48.14]	328 [53.59]
2006–2016	12,280 [23.68]	2799 [24.98]	1475 [19.30]	387 [30.79]	765 [45.13]	172 [28.10]
Comorbidities^2^, n (%)						
Cardiovascular disease	4817 [9.29]	1809 [16.14]	1093 [14.30]	212 [16.87]	369 [21.77]	135 [22.06]
Any Metabolic Comorbidity	3995 [7.70]	1894 [16.90]	980 [12.82]	259 [20.60]	499 [29.44]	156 [25.49]
• Diabetes	1141 [2.20]	987 [8.81]	473 [6.19]	125 [9.94]	266 [15.69]	123 [20.10]
• Obesity	79 [0.15]	158 [1.41]	79 [1.03]	21 [1.67]	37 [2.18]	21 [3.43]
• Hypertension	3285 [6.33]	1595 [14.23]	812 [10.63]	215 [17.10]	423 [24.96]	145 [23.69]
• Dyslipidemia	2071 [3.99]	815 [7.27]	387 [5.06]	117 [9.31]	244 [14.40]	67 [10.95]
Chronic Kidney Disease	186 [0.36]	125 [1.12]	70 [0.92]	16 [1.27]	31 [1.83]	8 [1.31]
Family history of early CVD	1859 [3.58]	291 [2.60]	196 [2.56]	39 [3.10]	43 [2.54]	13 [2.12]

All variables reported as mean (SD) or %, unless described otherwise. For definitions of the MASLD histological groups and all covariates, see the Appendix

MASLD, metabolic dysfunction-associated steatotic liver disease; MASH, metabolic dysfunction-associated steatohepatitis; CVD, cardiovascular disease; N., number; SD, standard deviation; IQR, interquartile range

^*^The index date was defined as the date of index liver biopsy (among patients with MASLD) or the corresponding matching date, among population controls, as per the Methods

^1^Education categories shown in the table were based on compulsory school, high school, and college (see eMethods). Education level was recorded beginning in 1990, thus data presented are for persons with index dates on or after January 1, 1990. For all other analyses, persons with index dates prior to 1990 had education level recorded as missing

^2^All covariates were defined as per Table S3. Any metabolic comorbidity was defined as: ≥ 1 metabolic risk factors (i.e. dyslipidemia, diabetes, hypertension and/or obesity). Family history of early CVD was defined as a recorded diagnosis of CVD in a first-degree family member before the age of 50 years (as per Additional file 1: Table S3)

### Overall arrhythmias

**Fig. 1:**
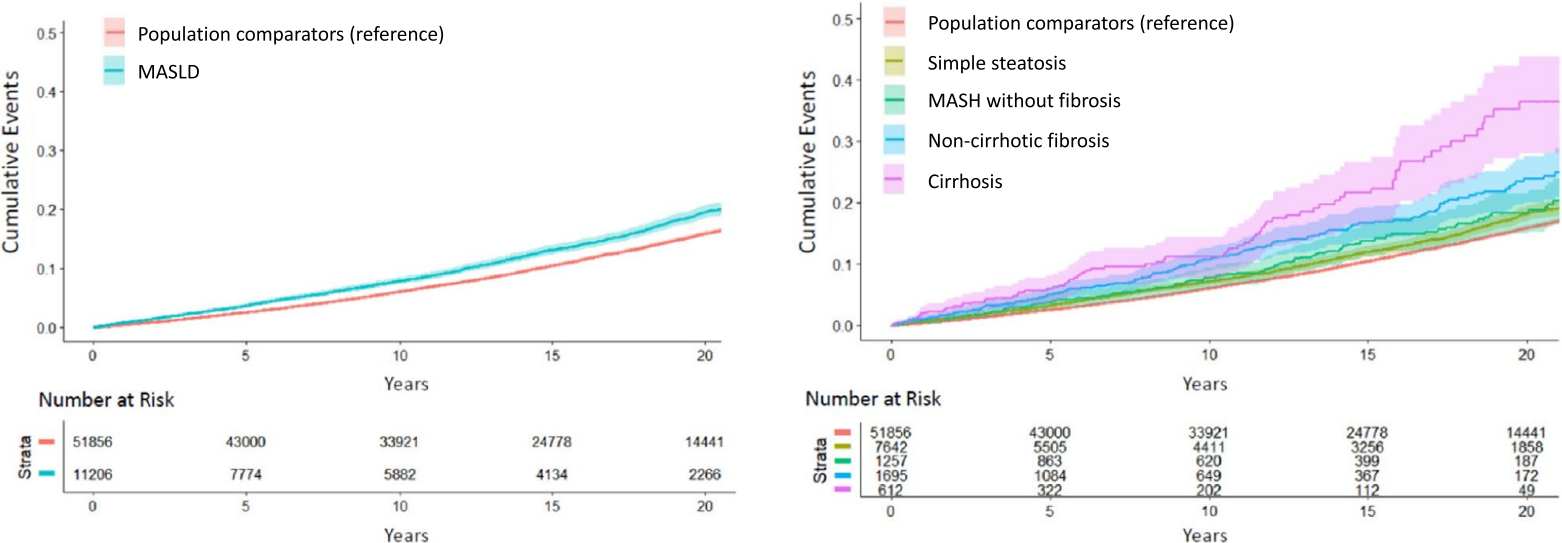
Cumulative Incidence of Arrhythmias According to the Presence and Histologic Severity of MASLD. *MASLD* metabolic dysfunction-associated steatotic liver disease; *MASH* metabolic dysfunction-associated steatohepatitis *Incident arrhythmias was a composite endpoint that included incident atrial fibrrillation or atrial flutter, bradyarrhythmias, other supraventricular arrhythmias, or ventricular arrhythmias or cardiac arrest (for definitions and details, see Additional file 1: Table S3)

Over 10.8 years of median follow-up, we confirmed 1,351 incident arrhythmias among MASLD patients (10.3/ 1000 person-years (PY)), and 6,493 incident arrhythmias among controls (8.7/1000PY; absolute rate difference, 1.7/1000PY, 95%CI 1.1–2.2)(Fig. 1; Table 2). After multivariable adjustment, MASLD patients had a significant, 1.30-fold higher rate of developing an incident arrhythmia, compared to controls (95%CI = 1.22–1.38), and significantly higher rates were observed across all MASLD histological categories (Fig. 1; Table 2). Specifically, compared to controls, the absolute rate differences and corresponding aHRs were significantly higher in patients with simple steatosis (1.1/1000PY; aHR = 1.28, 95%CI 1.19–1.38), non-fibrotic MASH (1.3/1000PY; aHR = 1.33, 95%CI 1.09–1.61), non-cirrhotic fibrosis (3.3/1000PY; aHR = 1.25, 95%CI 1.05–1.48) and cirrhosis (8.5/1000PY; aHR 1.60, 95%CI 1.21–2.12).

**Table 2:** Incident Overall Arrhythmias and Atrial Fibrillation among Adults with Histologically-Confirmed MASLD* and Matched Population Controls

	Population Controls (n = 51,856)	All MASLD (n = 11,206)	Simple Steatosis (n = 7642)	MASH without fibrosis (n = 1257)	Non-Cirrhotic Fibrosis (n = 1695)	Cirrhosis (n = 612)
Overall Arrhythmia						
N. of events	6493	1351	946	135	187	83
Incidence rate per 1000 PY (95% CI)	8.66 [8.45–8.87]	10.31 [9.77–10.86]	9.74 [9.14–10.37]	9.99 [8.44–11.74]	11.99 [10.39–13.76]	17.19 [13.88–21.09]
Absolute rate difference, per 1000 PY (95% CI)	0 [ref.]	1.65 [1.06–2.24]	1.08 [0.42–1.73]	1.33 [-0.37–3.02]	3.33 [1.6–5.06]	8.54 [4.83–12.24]
• Minimally-adjusted Model 1 (95% CI)	1 [ref.]	1.44 [1.36–1.53]	1.39 [1.29–1.49]	1.48 [1.23–1.79]	1.48 [1.26–1.74]	2.05 [1.61–2.61]
• Multivariable-adjusted Model 2 (95% CI)	1 [ref.]	1.30 [1.22–1.38]	1.28 [1.19–1.38]	1.33 [1.09–1.61]	1.25 [1.05–1.48]	1.60 [1.21–2.12]
Atrial fibrillation						
N. of events	5333	1055	735	105	154	61
Incidence rate per 1000 PY (95% CI)	7.05 [6.87–7.24]	7.96 [7.50–8.45]	7.48 [6.96–8.03]	7.71 [6.37–9.25]	9.80 [8.37–11.40]	12.37 [9.64–15.67]
Absolute rate difference, per 1000 PY (95% CI)	0 [ref.]	0.91 [0.39–1.42]	0.43 [-0.14–1]	0.66 [-0.83–2.14]	2.74 [1.18–4.3]	5.32 [2.21–8.43]
• Minimally-adjusted Model 1 (95% CI)	1 [ref.]	1.38 [1.30–1.48]	1.33 [1.23–1.44]	1.42 [1.15–1.76]	1.48 [1.24–1.76]	1.94 [1.47–2.57]
• Multivariable-adjusted Model 2 (95% CI)	1 [ref.]	1.26 [1.18–1.35]	1.24 [1.14–1.35]	1.34 [1.07–1.68]	1.24 [1.03–1.50]	1.59 [1.15–2.19]

*MASLD* metabolic dysfunction-associated steatotic liver disease; *MASH* metabolic dysfunction-associated steatohepatitis; *N* number; *PY* person years; *HR* hazard ratio; *CI* confidence interval; *ref* reference

^*^MASLD was defined by liver histology. Overall arrhythmia was a composite endpoint that included atrial fibrillation, bradyarrhythmias, other supraventricular arrhythmias and ventricular arrhythmias/cardiac arrest. For definitions of MASLD and arrhythmia outcomes, please see the Methods and the eMethods

^1^ Confidence intervals for incidence rates and absolute rate differences were approximated by the normal distribution. Incidence rate difference is per 1000 person years

^2^ The minimally-adjusted model 1 accounted for matching factors (age at the index date, sex, calendar year and county of residence). The fully-adjusted multivariable model accounted for the minimal model plus education, the number of recorded hospital visits in the 1 year prior to the index date (or corresponding matching date), and covariates defined up to and including the index date (i.e. diabetes, obesity, hypertension, dyslipidemia, chronic kidney disease and family history of cardiovascular disease before age 50 years), and alcohol use disorder during follow-up (defined as a time-varying covariate). For definitions, see the eMethods and Additional file 1: Table S3

In stratified analyses, the association between MASLD and incident overall arrhythmias was significantly higher in patients diagnosed with MASLD < 40 years of age (aHR = 2.22, 95%CI 1.80–2.74), compared to those diagnosed later in adulthood (aHR for 40–59 years = 1.30, 95%CI 1.18–1.42; aHR for >  = 60 years = 1.23, 95%CI 1.13–1.34; p-interaction < 0.001)( Additional file 1: Table S4). Otherwise, associations did not differ significantly by sex, duration of follow-up, or between patients with and without underlying metabolic comorbidities (p-interactions all > 0.05).

### Atrial fibrillation

Compared to controls, MASLD patients had modest but significantly higher rates of incident atrial fibrillation (difference, 0.9/1000PY; aHR = 1.26, 95%CI 1.18–1.35)(Table 2). These differences were observed across all MASLD histological categories, when compared to controls, including for simple steatosis (aHR = 1.24, 95%CI 1.14–1.35), non-fibrotic MASH (aHR = 1.34, 95%CI 1.07–1.68), non-cirrhotic fibrosis (aHR = 1.24, 95%CI 1.03–1.50) and cirrhosis (aHR = 1.59, 95%CI 1.15–2.19).

### Individual arrhythmia outcomes

Compared to controls, MASLD patients also had significantly higher rates of developing incident bradyarrhythmias (difference, 0.2/1000PY; aHR = 1.26, 95%CI 1.06–1.48), other supraventricular arrhythmias (difference, 0.1/1000PY; aHR = 1.27, 95%CI 1.00–1.62) and ventricular arrhythmias/cardiac arrest (difference, 0.6/1000PY; aHR = 1.53, 95%CI 1.30–1.80) (Additional file 1: Table S5). In further exploratory analyses, we assessed those individual arrhythmia outcomes according to MASLD histological categories (Additional file 1: Table S5); however, those findings merit cautious interpretation, given the small numbers in each subgroup.

### MASLD-only subgroup

When compared to patients with simple steatosis, those with more advanced MASLD fibrosis had significantly higher rates of incident arrhythmias (absolute differences for non-cirrhotic fibrosis and for cirrhosis, 2.3/1000PY and 7.5/1000PY, respectively; Table 3). After multivariable adjustment, this corresponded to a 32% higher rate of incident arrhythmias in patients with cirrhosis, compared to those with simple steatosis (aHR = 1.32, 95%CI 1.04–1.66). Findings were similar in analyses focused on incident atrial fibrillation (differences for non-cirrhotic fibrosis and cirrhosis, each compared to simple steatosis: 2.3/1000PY and 7.5/1000PY, respectively). In contrast, no significant differences in either overall arrhythmias or in atrial fibrillation were observed, when non-fibrotic MASH was compared directly with simple steatosis.

**Table 3:** Incident overall arrhythmias and atrial fibrillation in the MASLD-only subgroup, with simple steatosis as the comparator

	Simple Steatosis (reference group) (n = 7642)	MASH without fibrosis (n = 1257)	Non-Cirrhotic Fibrosis (n = 1695)	Cirrhosis (n = 612)
Overall Arrhythmias				
N. of events	946	135	187	83
Incidence rate per 1000 PY (95% CI)	9.74 [9.14–10.37]	9.99 [8.44–11.74]	11.99 [10.39–13.76]	17.19 [13.88–21.09]
Absolute rate difference, per 1000 PY (95% CI)	0 [ref.]	0.25 [-1.55–2.04]	2.25 [0.42–4.07]	7.46 [3.71–11.21]
Minimally-adjusted Model 1 (95% CI)	1 [ref.]	1.03 [0.85–1.24]	1.10 [0.93–1.30]	1.48 [1.18–1.86]
Multivariable-adjusted Model 2 (95% CI)	1 [ref.]	1.01 [0.84–1.21]	1.07 [0.90–1.26]	1.31 [1.04–1.66]
Atrial Fibrillation				
N. of events	735	105	154	61
Incidence rate per 1000 PY (95% CI)	7.48 [6.96–8.03]	7.71 [6.37–9.25]	9.80 [8.37–11.40]	12.37 [9.64–15.67]
Absolute rate difference, per 1000 PY (95% CI)	0 [ref.]	0.23 [-1.34–1.8]	2.31 [0.68–3.95]	4.89 [1.74–8.04]
Minimally-adjusted Model 1 (95% CI)	1 [ref.]	1.02 [0.82–1.26]	1.18 [0.98–1.41]	1.39 [1.06–1.81]
Multivariable-adjusted Model 2 (95% CI)	1 [ref.]	1.01 [0.81–1.24]	1.15 [0.95–1.38]	1.25 [0.95–1.64]

*MASLD* metabolic dysfunction-associated steatotic liver disease; *MASH* metabolic dysfunction-associated steatohepatitis; *N* number; *PY* person years; *HR* hazard ratio; *CI* confidence interval; *ref.* referent

^1^Confidence intervals for incidence rates and absolute rate differences were approximated by the normal distribution. Incidence rate difference is per 1000 person years

^2^The minimally-adjusted model 1 accounted for matching factors (age at the index date, sex, calendar year and county of residence). The fully-adjusted multivariable model accounted for the minimal model plus education, the number of recorded hospital visits in the 1 year prior to the index date (or corresponding matching date), and covariates defined up to and including the index date (i.e. diabetes, obesity, hypertension, dyslipidemia, chronic kidney disease and family history of cardiovascular disease before age 50 years), and alcohol use disorder during follow-up (defined as a time-varying covariate). For definitions, see the eMethods and Additional file 1: Table S3

### Sibling analyses

To address potential residual confounding related to shared genetic and intrafamilial factors, we re-matched 4,686 MASLD patients to 8,736 full-sibling comparators who were alive at the index date and had no diagnoses of MASLD or arrhythmia (Additional file 1: Table S6). Consistent with our primary analysis, MASLD patients again demonstrated significantly higher rates of incident overall arrhythmias, compared to matched full siblings (difference, 2.3/1000PY; aHR = 1.34, 95%CI 1.15–1.56), including higher rates of incident atrial fibrillation (difference, 1.6/1000PY; aHR = 1.32, 95%CI 1.11–1.58). In analyses of the individual arrhythmia outcomes, we found that MASLD patients had significantly higher rates of incident ventricular arrhythmias/cardiac arrest, compared to full sibling controls (difference, 0.6/1000PY; aHR = 1.89, 95%CI 1.25–2.86), but no significant differences were observed for bradyarrhythmias or other supraventricular arrhythmias.

### Sensitivity Analyses

Our findings were robust across all sensitivity analyses, including after restricting the cohort to MASLD patients with index biopsy ≥ January 1, 2006 (n = 2375) and matched controls (n = 9757), and further adjusting for use of relevant medications (difference_overall arrhythmias_ for MASLD vs. controls = 3.5/1000PY; aHR = 1.47, 95%CI 1.20–1.81]; Additional file 1: Table S7). Similarly, our findings persisted after further adjusting for COPD as a proxy for smoking (aHR_overall arrhythmias_ = 1.29, 95%CI 1.21–1.37), and also after excluding any patient with a cardiac arrhythmia outcome within < 90 days of follow-up (aHR_overall arrhythmias_ = 1.28, 95%CI 1.20–1.36; Additional file 1: Table S8) or within < 2 years (aHR_overall arrhythmias_ = 1.27; 95%CI 1.19–1.36; Additional file 1: Table S9). Moreover, our results were similar in analyses focused on primary cardiac arrhythmias (i.e. after excluding any patient with underlying CVD at baseline, and further censoring patients with incident CVD diagnosed during follow-up: aHR_overall arrhythmias_ = 1.29, 95%CI 1.19–1.40 Additional file 1: Table S10). Finally, we estimated the “E-value” using the approach by VanderWeele et al [41], and found that an unmeasured confounder would need to have a multivariable-adjusted HR of at least 1.92 or more for both the exposure and outcome, to attenuate the observed association between MASLD and incident arrhythmias to 1.0. For cirrhosis, this E-value would need to be at least 2.58 or more.

## Discussion

In this population-based cohort comprised of all Swedish adults with biopsy-confirmed MASLD and matched general population controls, MASLD was associated with significantly higher rates of both fatal and non-fatal cardiac arrhythmias, including atrial fibrillation, bradyarrhythmias, other supraventricular arrhythmias and ventricular arrhythmias/cardiac arrest. Compared to controls, MASLD patients had a 30% higher relative risk of developing an arrhythmia, and an absolute excess rate of 1.7 per 1000PY, which corresponds to one additional, incident cardiac arrhythmia per each 29 patients diagnosed with MASLD over 20 years. Rates of incident overall arrhythmias and atrial fibrillation were significantly elevated across all MASLD histological categories, and they were highest in patients with cirrhosis, who demonstrated striking, 17-fold higher rates of developing an incident cardiac arrhythmia—including 12-fold higher rates of incident atrial fibrillation—compared to controls. Importantly, these findings were robust across numerous sensitivity analyses, including after accounting for cardiometabolic risk factors, use of relevant medications, other causes of death, and after comparing MASLD patients with full siblings, to address important, shared genetic, intrafamilial and socioeconomic factors.

Currently, longitudinal evidence linking MASLD to the development of incident cardiac arrhythmias, including atrial fibrillation, is limited. While a recent meta-analysis of six longitudinal studies found a significant, 19% higher risk of incident atrial fibrillation in patients with MASLD compared to non-MASLD controls (pooled HR = 1.19, 95%CI 1.07–1.31) [18], a prior meta-analysis and recent observational community-based cohort [17, 22] found null associations. However, all prior studies have been significantly limited by small sample sizes, cross-sectional design [24], or reliance upon surrogate definitions of MASLD, including laboratory-based algorithms [42], ultrasound [20, 21] or computed tomography [22], which lack precision and cannot identify MASH or stage fibrosis. In fact, the largest studies to date have identified people with MASLD using the fatty liver index—an algorithm based on BMI, waist circumference, triglycerides and GGT—which is not specific for MASLD but rather reflects the association with obesity. In contrast, the current study derived from a large, nationwide cohort with detailed liver histology, and long follow-up for incident arrhythmia events. Thus, our data provide strong evidence that MASLD is associated with modest albeit significantly increased rates of developing incident arrhythmias, including atrial fibrillation.

Currently, little is known about the impact of MASLD histological severity on risk of developing incident cardiac arrhythmias [17]. In the current study, we found that the incidence of arrhythmias including atrial fibrillation were significantly elevated across all MASLD histological groups, including in patients with earlier stages of non-fibrotic MASLD. Nevertheless, among patients with MASLD, we found that patients with non-cirrhotic fibrosis or cirrhosis had markedly higher absolute rates of developing incident arrhythmias, when compared to simple steatosis (absolute differences, 2.3 and 7.5 per 1000PY, respectively). Over 20 years, those rate differences correspond to one additional, incident cardiac arrhythmia diagnosis per each 22 patients with non-cirrhotic fibrosis, and one additional arrythmia diagnosis per each 7 patients with cirrhosis. In contrast, rates of incident arrhythmias did not appear to differ between patients with non-fibrotic MASH and simple steatosis. Collectively, these data suggest that advanced MASLD fibrosis is an important predictor of incident arrhythmias, including atrial fibrillation.

Although the precise mechanism is undefined, preclinical and preliminary clinical studies suggest that MASLD may contribute to cardiac arrhythmia risk by promoting cardiac remodeling [10], autonomic dysfunction [43–45], and/or electrical remodeling [46]. For example, MASLD has been linked to cardiac sympathetic/parasympathetic dysregulation [47], impaired heart rate variability [45], and also to QTc interval [48–50] and cardiac conduction abnormalities [48, 51], and a higher risk of recurrent atrial fibrillation after ablation [52]. Furthermore, advanced liver disease at the stage of cirrhosis is known to considerably impair cardiac function via a hyperdynamic circulation and vasodilation, what has been termed “cirrhotic cardiomyopathy” [53]. In addition, MASLD represents a complex, multi-system disease, and it is still unclear whether MASLD represents a causal driver of cardiac arrhythmias, or whether it might simply reflect other, shared cardiometabolic risk factors. Importantly, we lacked data on BMI for the current study, which is an established risk factor for both MASLD and atrial fibrillation; however, evidence suggests that each 5-unit increase in BMI above the normal range confers a 19%-29% increased relative risk of incident atrial fibrillation [54]. Thus, our sensitivity analysis for unmeasured confounding (E-value = 1.92) would suggest that the association between MASLD and incident arrhythmias is unlikely to be fully explained by obesity; nevertheless, future large-scale cohorts with detailed assessments of adiposity are needed, to fully characterize this relationship.

Strengths of this study include a large, nationwide population with comprehensive and prospectively-recorded histopathology data, and strict, validated definitions of both MASLD [12] and confounding variables, in registers with near-complete follow-up for the entire Swedish population. Our large sample size permitted calculation of more precise risk estimates across the MASLD histological spectrum, which was not possible in previous, smaller studies, and allowed us to account for cardiometabolic risk factors and potential confounders. We also applied robust techniques to address potential residual confounding, misclassification of non-MASLD controls, and potential competing events.

### Study limitations

We acknowledge several limitations. First, this was a retrospective study of biopsy-confirmed MASLD, which could introduce selection bias. However, our risk estimates are highly consistent with the largest meta-analysis to date [18], which was comprised of smaller cohorts that defined MASLD using non-invasive approaches, highlighting the generalizability of our findings. Second, while it is possible that our controls could have included patients with undiagnosed MASLD, our findings were consistent in the MASLD-only subgroup. Third, despite careful matching, residual confounding is possible, and we lacked detailed laboratory data or information regarding smoking status, alcohol consumption, or BMI. Nevertheless, our findings were robust after multivariable adjustment for important risk factors, including clinical comorbidities, subsequent alcohol use disorders and COPD − as a proxy for heavy smoking, relevant medication use, and even a first-degree family history of cardiovascular disease. Moreover, after re-matching MASLD patients with full siblings to address shared intrafamilial and early-life factors, the associations were confirmed. Finally, the Swedish population is primarily Caucasian, highlighting the need for additional research in more diverse, large-scale histology cohorts.

In conclusion, within this large nationwide cohort of 11,206 adults with biopsy-confirmed MASLD and matched population controls, MASLD was associated with modest but significantly higher incidence of both fatal and non-fatal arrhythmias, including atrial fibrillation, bradyarrhythmias, other supraventricular arrhythmias and ventricular arrhythmias/cardiac arrest. Our study provides quantitative estimates regarding rates of incident arrhythmias according to the presence and histological severity of MASLD, for the first time on a nationwide scale. The incidence of arrhythmias, including atrial fibrillation, was significantly elevated across all stages of MASLD, particularly in cirrhosis. Thus, our results provide strong support for additional research to understand the mechanisms that link MASLD and fibrosis to the development of arrhythmias, and also to identify which high-risk patients with MASLD might benefit from increased surveillance or early interventions.

### Supplementary Information

**Additional file 1: Table S1.** Study Exclusion Criteria.** Table S2.** Definitions of Primary and Secondary Outcomes.** Table S3.** Definitions of Covariates and Prescription Medications.** Table S4.** Stratified Models for the Primary Overall Arrhythmia Outcome.** Table S5.** Secondary Arrhythmia Outcomes among Adults with Histologically-Confirmed MASLD and Matched Population Controls.** Table S6.** Incident Overall Arrhythmias and Atrial Fibrillation among MASLD Patients and Matched Full Sibling Comparators.** Table S7.** Incident Arrhythmia Outcomes among Adults with Histologically-Confirmed MASLD and Matched Population Controls from 2006-2016 with Comprehensive Prescription Medication Use Data.** Table S8.** Incident Cardiac Arrhythmias among MASLD Patients and Matched Population Controls, after Excluding Patients with a Primary Outcome Within <90 Days.** Table S9.**Incident Cardiac Arrhythmias among MASLD Patients and Matched Population Controls, after Excluding Patients with a Primary Outcome Within <2 years.** Table S10.** Incident Primary Cardiac Arrhythmias among MASLD Patients and Matched Population Controls, after Excluding Patients with Underlying CVD and Censoring at the Date of CVD Diagnoses in Follow-up.** Figure S1.** Cohort Construction

## Data Availability

No additional data are available due to Swedish regulations.

## Abbreviations

aHR: Adjusted hazard ratio
AF: Atrial fibrillation
CV: Cardiovascular
CVD: Cardiovascular disease
CI: Confidence interval
ESPRESSO: Epidemiology Strengthened by histoPathology Reports in Sweden
ICD: International classification of diseases
MASLD: Metabolic dysfunction-associated steatotic liver disease
MASH: Metabolic dysfunction-associated steatohepatitis
NAFLD: Nonalcoholic fatty liver disease
NASH: Nonalcoholic steatohepatitis
PPV: Positive predictive value
PY: Person-Years
