# Supplementary material for: Incident cardiac arrhythmias associated with metabolic dysfunction-associated steatotic liver disease: a nationwide histology cohort study
Source: Cardiovasc Diabetol. 2023 Dec 13;22:343. doi: 10.1186/s12933-023-02070-5 (PMC10720135; doi:10.1186/s12933-023-02070-5)
Supplement: Supplementary file 1 — Additional file 1: Table S1. Study Exclusion Criteria. Table S2. Definitions of Primary and Secondary Outcomes. Table S3. Definitions of Covariates and Prescription Medications. Table S4. Stratified Models for the Primary Overall Arrhythmia Outcome. Table S5. Secondary Arrhythmia Outcomes among Adults with Histologically-Confirmed MASLD and Matched Population Controls. Table S6. Incident Overall Arrhythmias and Atrial Fibrillation among MASLD Patients and Matched Full Sibling Comparators. Table S7. Incident Arrhythmia Outcomes among Adults with Histologically-Confirmed MASLD and Matched Population Controls from 2006-2016 with Comprehensive Prescription Medication Use Data. Table S8. Incident Cardiac Arrhythmias among MASLD Patients and Matched Population Controls, after Excluding Patients with a Primary Outcome Within <90 Days. Table S9.Incident Cardiac Arrhythmias among MASLD Patients and Matched Population Controls, after Excluding Patients with a Primary Outcome Within <2 years. Table S10. Incident Primary Cardiac Arrhythmias among MASLD Patients and Matched Population Controls, after Excluding Patients with Underlying CVD and Censoring at the Date of CVD Diagnoses in Follow-up. Figure S1. Cohort Construction [file 12933_2023_2070_MOESM1_ESM.docx]

**Additional Appendix**

*eMethods*

- Definition of MASLD and MASLD Histological Categories
- Definitions of Outcomes and Covariates
- Missing Data

*eTables*

- **Table S1.** Study Exclusion Criteria
- **Table S2.** Definitions of Primary and Secondary Outcomes
- **Table S3.** Definitions of Covariates and Prescription Medications
- **Table S4.** Stratified Models for the Primary Overall Arrhythmia Outcome
- **Table S5.** Secondary Arrhythmia Outcomes among Adults with Histologically-Confirmed MASLD and Matched Population Controls
- **Table S6.** Incident Overall Arrhythmias and Atrial Fibrillation among MASLD Patients and Matched Full Sibling Comparators
- **Table S7.** Incident Arrhythmia Outcomes among Adults with Histologically-Confirmed MASLD and Matched Population Controls from 2006-2016 with Comprehensive Prescription Medication Use Data
- **Table S8.** Incident Cardiac Arrhythmias among MASLD Patients and Matched Population Controls, after Excluding Patients with a Primary Outcome Within <90 Days
- **Table S9.** Incident Cardiac Arrhythmias among MASLD Patients and Matched Population Controls, after Excluding Patients with a Primary Outcome Within <2 years
- **Table S10.** Incident Primary Cardiac Arrhythmias among MASLD Patients and Matched Population Controls, after Excluding Patients with Underlying CVD and Censoring at the Date of CVD Diagnoses in Follow-up

*Supplementary Figures*

- **Figure S1.** Cohort Construction

*Additioanal References*

**eMethods:**

*Definition of MASLD and MASLD Histological Categories*

Consistent with nationwide liver histopathology reporting recommendations provided to all pathologists in Sweden^1^, we defined MASLD from histopathology data using an established algorithm of SNOMED topography and morphology codes, after excluding other etiologies of liver disease. Specifically, patients were identified by a liver biopsy histopathology report that included a topography code for liver (T56), and at least one morphology code for steatosis (M5008x or M5520x), consistent with our validation study in ESPRESSO (see below). In Sweden, clinically-indicated liver biopsies are conducted with a single pass of the liver, unless a satisfactory specimen is not obtained with the initial pass^1^.

Among individuals with multiple liver biopsies that demonstrated steatosis, we included the first such biopsy. We excluded anyone with at least one recorded diagnoses of another etiology of liver disease (all outlined in **Table S1**; i.e. alcohol-related liver disease, drug-induced liver disease, viral hepatitis B or C infection, Budd-Chiari, liver abscess, HIV/AIDS, autoimmune hepatitis, primary biliary cholangitis, other cholangitis, alpha-1 antitrypsin deficiency, glycogen storage disease), or a recorded diagnosis of alcohol abuse/misuse or drug abuse, or a recorded diagnosis of a cardiac arrhythmia (see **Table S1**). Additionally, we further excluded anyone with use of any steatogenic medication or use of a medication specific for the treatment of an alternative etiology of liver disease, or use of an anti-arrhythmic medication (as per **Table S1**). Medication use was defined by the Prescribed Drug Register as a filled prescription for at least 30 cumulative defined daily doses (cDDD) of a given medication, at any time prior to the index date (or corresponding matching date), with the exception of systemic steroids (for which a person was excluded if they used systemic steroids within 0 to 3 months prior to the index date).

*Definitions of Outcomes and Covariates*

The two primary outcomes were: (1) incident overall arrhythmias (a composite outcome defined by ≥1 primary or secondary inpatient or outpatient ICD diagnosis for atrial fibrillation or atrial flutter, bradyarrhythmia, other supraventricular arrhythmia, ventricular arrhythmias or cardiac arrest), and (2) incident atrial fibrillation. The definitions of each primary and secondary outcome are outlined in the Methods and in **Table S2**, below.

Definitions of clinical, demographic and prescription medication covariates are outlined in the **Methods** and in **Table S3**. Since 1990, the longitudinal integrated database for health insurance and labour market studies (LISA) database^3^ has prospectively recorded and annually-updated detailed data from the Swedish labor market, and from the educational and social sectors, for all Swedish residents aged 16 years and older. Education level, a proxy for socioeconomic status, was obtained from the validated LISA database^3^.

For the primary analysis, our main multivariable model adjusted for matching factors (i.e. age at the index date/corresponding matching date, sex, calendar year and county in Sweden) and *a priori-*selected prognostic covariates, as outlined in the **Methods**. In an additional sensitivity analysis, we repeated the primary analysis after restricting the cohort to persons with an index date on or after January 1, 2006, and then constructed an additional multivariable-adjusted model that further accounted for *a priori* selected medication covariates (i.e., low-dose aspirin [<163mg dosage], other antiplatelets, statins, antidiabetic medications, anti-hypertensive medications and anticoagulants, all defined in **Table S3**). Use of each medication was defined from the Prescription Drug Register as at least 30 cumulative defined daily doses (cDDD) of filled prescriptions for that medication from a Swedish pharmacy.

**Table S1.** Study Exclusion Criteria*

| **Excluded Conditions^1^:** | **ICD-7 / 8** | **ICD-9** | **ICD-10** |
| --- | --- | --- | --- |
| Alcohol abuse / misuse, or  Alcohol-related liver disease | 280,00; 281,00; 307,00; 307,10; 307,99; 322; 581,10; 583,10; 261,00; 262,00; 291; 291,1; 303; 571,00; 571,01; 979; 980,00; 980,01; 980,98; 980,99 | 291; 294A; 303; 305A; 357F; 425F; 535D; 571A-D; 760W; 790D; 977D; 980A; 980X; V97B | E24.4; F10; G31.2; G62.1; G72.1; I42.6; K29.2; K70; K86.0; Q35.4; R78.0; T51.0; T51.8; T51.9; X65; Y15; Y57.3; Y90; Y91; Z50.2; Z71.4; Z71.2 |
| Other drug abuse | 5710, E860, N980 | 571A-D | F11-F19 |
| Drug-induced liver disease |  | 573D | K71 |
| Viral hepatitis B or C infection |  | 70; 070; ICD-8: 070; 999,20 | B15-19, B16.0, B16.1, B16.2, B16.9, B17.0-B17.9, B18.0-B18.9, B19.0-B19.9; B00.8; B25.1 |
| Budd-Chiari |  | 453A | I82 |
| Liver abscess | 572; 572,00 | 5720 | K75.0, A06.4 |
| HIV |  | 279K | B20-B24 |
| Hemochromatosis | 273,2 | 275A | E83.1 |
| Wilson’s disease | 273,3 | 275B | E83.0 |
| Autoimmune hepatitis |  | 571.42 | K75.4 |
| Primary biliary cholangitis |  | 571G | K74.3, K74.4 |
| Other cholangitis | 574,06 | 576B | K83; K83.0A |
| Alpha-1 antitrypsin deficiency |  | 273.4; 277G; 274E; 573W | E88.01 |
| Glycogen storage disease |  | 271.8 | E74.09, E74.00 |
| Cardiac arrhythmia | See Table S2 | See Table S2 | See Table S2 |
| **Excluded Medications^2^:** | | **Prescribed Drug Register ATC Code:** | |
| Systemic steroids | | 01AC02, C05AA09, D07AB19, D10AA03, H02AB02, R01AD03, S01BA01, S01CB01, S02BA06, S03BA01, H02AB15, A01AC03, A07EA02, C05AA01, D07AA02, D07XA01, H02AB09, S01BA02, S01CB03, S02BA01, D07AC16, D07AB11, D07AB02, D07AC16, A07AE03, H02AB07 | |
| Tamoxifen | | L02BA01 | |
| Methotrexate | | L01BA01, L04AX03 | |
| Interferon | | L03AB | |
| Direct-acting antiviral therapy | | JO5, JO5A, JO5AA-AH, JO5AP, JO5AR | |
| Nucleos(t)ide reverse transcriptase inhibitors / NNRTIs | | J05AF, J05AG | |
| Valproic acid | | N03AG01 | |
| Amiodarone | | C01BD01 | |
|  | |  | |
| Antiarrhythmics | | C01B | |
|  | |  | |
|  | |  | |

Abbreviations: ICD, International Classification of Disease; ATC, anatomic therapeutic chemical classification

*For study flowchart outlining exclusion criteria, please see Figure S1

^1^ We excluded any person with a first recorded primary diagnosis or the second of two secondary diagnoses for a cardiac arrhythmia, or another etiology of liver disease, or alcohol abuse/misuse or alcohol-related liver disease, defined on or prior to the index date.

^2^We excluded any person with use of a steatogenic medication or a medication used to treat an alternative etiology of liver disease, or a medication used to treat a cardiac arrhythmia, on or prior to the index date. Medication use was defined by a filled prescription for at least 30 cumulative defined daily doses (cDDD), in the Prescribed Drug Register, at any time prior to the index date (or corresponding matching date), with the exception of systemic steroids (for which a person was excluded if they used systemic steroids within 0 to 3 months prior to the index date). As the Prescribed Drug Register began on July 1, 2005, these exclusion criteria were only applied to the subgroup analysis in which the cohort was restricted to persons with an index biopsy (or corresponding matching date) on or after January 1, 2006.

**Table S2.** Definitions of Primary and Secondary Outcomes

**Outcome Source Definition^a^**

Overall Arrhythmia Hospital discharge letters or outpatient Composite outcome including any of the

specialty care letters following individual outcomes: (1) atrial fibrillation or atrial flutter, (2) bradyarrhythmias, (3) other supraventricular arrhythmias, and (4) ventricular arrhythmias / cardiac arrest (each defined below)

Atrial fibrillation / flutter Hospital discharge letters or outpatient ICD-8: 427.92 specialty care letters ICD-9: 427D

ICD-10: I48

Bradyarrhythmias Hospital discharge letters or outpatient ICD-8: 427.20; 427.27-9

specialty care letters ICD-9: 426A-B; 426G; 426X

Other supraventricular arrhythmias Hospital discharge letters or outpatient ICD-8: 427.90

specialty care letters ICD-9: 426H; 427A

ICD-10: I45.6; I47.1

Ventricular arrhythmias / cardiac arrest Hospital discharge letters or outpatient ICD-8: 427.91, 795.99

specialty care letters ICD-9: 427B; 427D-F; 798B-C;

ICD-10: I46.0-1; I46.9; I47.0; I49.0; R96.0

**Table S3.** Definitions of Covariates and Prescription Medications

**Entity Source Definition^a^**

Low-dose aspirin^c^ Prescribed Drug Register^b^ Medication ATC code B01AC06

Statins Prescribed Drug Register Medication ATC codes: C10

Metformin Prescribed Drug Register Medication ATC code: A10BA02

Insulin Prescribed Drug Register Medication ATC codes: A10A

Other glucose-lowering agents Prescribed Drug Register Medication ATC codes: A10B (excluding

(not metformin or insulin) A10BA02)

Antihypertensive medications Prescribed Drug Register Medication ATC codes: C02A-N, C03AA-AB,

C03BA, C03CA, C03DA, C03EA, C08DA51,

C08DA, C08CA, C08DA, C08DB, C09AB

C09BA-BB, C09CA-CB, C09DA, C09DB01

Vitamin K antagonists Prescribed Drug Register Medication ATC codes: B01AA

Direct thrombin inhibitors Prescribed Drug Register Medication ATC codes: B01AE

Direct factor Xa inhibitors Prescribed Drug Register Medication ATC codes: B01AF

Other antithrombotic medications Prescribed Drug Register Medication ATC codes: B01AX

Coronary vasodilators Prescribed Drug Register Medication ATC codes: C07AA, C07AB,

C07AB02, C07BB, C07CB, C07AG01

Liver transplantation Hospital discharge letters, using the Codes: 5200, 5202, JJC00, JJC10, JJC20,

Swedish Classification of Operation and JJC30, JJC40

Major Procedure codes

Chronic kidney disease Hospital discharge letters or outpatient ICD-8: 585, 586, Y29,01

specialty care letters (Patient Register) ICD-9: 585, 586, 753B, V42A, V45B, V56

ICD-10: N18-23; N26; T82.4; Y84.1; Q61; Z49; Z99.2; Z94.0

**Entity Source Definition^a^**

Hypertension Hospital discharge letters or outpatient ICD-9: 400-404

specialty care letters (Patient Register) ICD-9: 401-405

and/or receipt of at least one 30-day filled ICD-10: I10-I16

prescription for an anti-hypertensive drug Medication ATC codes: (see

(Prescribed Drug Register) antihypertensives, above)

Diabetes Hospital discharge letters containing ICD-8 / ICD-9: 250

either a primary or a secondary ICD-10: E10.0-E14.9

diagnosis for diabetes or outpatient specialty Medication ATC Codes: A10A (insulin),

care diagnosis (Patient Register), and/or at A10BA (biguanides),

least one 30-day filled prescription for an A10BB-BX (all other antidiabetic drugs)

antidiabetic agent (Prescribed Drug Register)

Obesity Hospital discharge letters or outpatient ICD-8/9: 278, 649,1, 649B

specialty care letters ICD-10: E65-66

Dyslipidemia Hospital discharge letters or outpatient ICD-8: 272

specialty care letters (Patient Register) ICD-9: 272

and/or receipt of at least one 30-day filled ICD-10: E78

prescription for a statin medication Medication ATC codes: C10

(Prescribed Drug Register)

Cardiovascular disease (CVD) Hospital discharge letters or outpatient See definitions of each, below

specialty care letters (Patient Register) for

a diagnosis of ischemic heart disease,

congestive heart failure, or stroke or transient

ischemic attack (each defined below)

Ischemic Heart Disease Hospital discharge letters or outpatient ICD-8/9: 410-414

specialty care letters ICD-10: I20-25

Congestive Heart Failure (CHF) Hospital discharge letters or outpatient ICD-8: 427,00; 427,10

specialty care letters ICD-9: 428A; 428B; 428X

ICD-10: I11.0; I50

Stroke Hospital discharge letters or outpatient ICD-8/9: 430-438

specialty care letters ICD-10: I60-64

Family history of cardiovascular disease in a first-degree Hospital discharge letters or outpatient See above (Table S3) for CVD definitions

family member, before the age of 50 years specialty care letters (Patient Register) or a

primary cause of death (Cause of Death Register)

that meet criteria for a MACE outcome

Chronic Obstructive Pulmonary Disease (COPD) Hospital discharge letters or outpatient ICD-8/9: 490-496

Specialty care letters (Patient Register) ICD-10: J40-47

Abbreviations: ICD, International Classification of Diseases; ATC, Anatomical Therapeutic Chemical classification system

^a^Covariates were defined using ICD classifications. Note that ICD 4th letter codes are used in Swedish versions of ICD.

^b^The Prescribed Drug Register includes dates of prescriptions and dispensations, defined daily doses (DDD) of all drug prescriptions and dispensations, according to the ATC classification System. For covariates that included medications, relevant medication use was defined as the first filled prescription for >30 cDDD.

^c^Low-dose aspirin prescriptions in Sweden include strengths of <163mg (typically either 75mg or 160mg)

**Table S4.** Stratified Models for the Primary Overall Arrhythmia Outcome*

|  | **N. of cases / Person-Years** | **Adjusted HR (95% CI)** | **P-Interaction** |
| --- | --- | --- | --- |
| **Overall Arrhythmia*** | | |  |
| Male | 751/76.13 | 1.29 [1.19-1.40] |  |
| Female | 600/54.96 | 1.37 [1.25-1.51] | 0.32 |
|  |  |  |  |
| Age at index biopsy |  |  |  |
| 18-39 years | 130/37.24 | 2.22 [1.80-2.74] |  |
| 40-59 years | 584/65.28 | 1.30 [1.18-1.42] |  |
| ≥60 years | 637/28.57 | 1.23 [1.13-1.34] | <0.001 |
|  |  |  |  |
| Duration of follow-up (years) |  |  |  |
| <2 years | 148/19.80 | 1.57 [1.30-1.90] |  |
| 2 to <10 years | 490/59.33 | 1.37 [1.24-1.52] |  |
| ≥10 years | 713/51.96 | 1.28 [1.19-1.38] | 0.12 |
|  |  |  |  |
| Index biopsy year |  |  |  |
| 1969-1989 | 333/30.57 | 1.17 [1.04-1.33] |  |
| 1990-2005 | 881/87.93 | 1.32 [1.23-1.43] |  |
| 2006-2017 | 137/12.59 | 1.30 [1.06-1.59] | 0.26 |
|  |  |  |  |
| Any metabolic comorbidity | 112/6.73 | 1.09 [0.69-1.71] |  |
| No metabolic comorbidity | 1239/124.36 | 1.33 [1.25-1.41] | 0.40 |
|  |  |  |  |
| Hypertension | 152/7.96 | 1.27 [0.95-1.69] |  |
| No hypertension | 1199/123.13 | 1.33 [1.25-1.42] | 0.75 |
|  |  |  |  |
| Dyslipidemia | 64/3.54 | 0.82 [0.49-1.38] |  |
| No dyslipidemia | 1287/127.55 | 1.31 [1.23-1.39] | 0.08 |
|  |  |  |  |
| Diabetes | 110/5.57 | 0.77 [0.34-1.75] |  |
| No Diabetes | 1241/125.52 | 1.31 [1.23-1.39] | 0.21 |
|  |  |  |  |
| Family history of CVD <age 50 years | 29/3.83 |  |  |
| Family history of CVD ≥age 50 years | 1322/127.26 | 1.32 [1.24-1.40] | 0.90 |
|  |  |  |  |

Abbreviations: CVD, cardiovascular disease; N., number; HR, hazard ratio; CI, confidence interval

*MASLD was defined by histology. For definitions of MASLD and arrhythmia outcomes, please see the Methods and eMethods.

The multivariable-adjusted model accounted for matching factors (age at the index date, sex, calendar year and county of residence), education level, the number of recorded hospital encounters in the 1 year prior to the index date, and the following covariates: diabetes (yes/no), obesity (yes/no), hypertension (yes/no), dyslipidemia (yes/no), chronic kidney disease (yes/no), family history of cardiovascular disease before age 50 years (yes/no) and alcohol use disorder (yes/no – defined as a time-varying covariate, updated over study follow-up).

All covariates were defined as per Table S3. Any metabolic comorbidity was defined as: ≥1 metabolic risk factor (i.e. dyslipidemia, diabetes, hypertension and/or obesity).

**Table S5.** Secondary Arrhythmia Outcomes among Adults with Histologically-Confirmed MASLD* and Matched Population Controls

|  | **Population Controls**  **(n=51,856)** | **All MASLD**  **(n=11,206)** | **Simple Steatosis**  **(n=7,642)** | **MASH without fibrosis**  **(n=1,257)** | **Any Fibrosis**  **(n=2,307)** |
| --- | --- | --- | --- | --- | --- |
| **Bradyarrhythmia** | | | | |  |
| N. of events | 923 | 183 | 135 | 15 | 33 |
| Incidence rate per 1000 PY (95% CI) | 1.18 [1.11-1.26] | 1.33 [1.16-1.54] | 1.33 [1.12-1.56] | 1.07 [0.65-1.67] | 1.54 [1.10-2.10] |
| Absolute rate difference, per 1000 PY (95% CI) | 0 [ref.] | 0.15 [-0.06-0.36] | 0.15 [-0.09-0.38] | -0.12 [-0.66-0.43] | 0.35 [-0.18-0.88] |
| - Minimally-adjusted Model 1 (95% CI) | 1 [ref.] | 1.40 [1.19-1.64] | 1.48 [1.22-1.78] | 0.93 [0.54-1.61] | 1.36 [0.93-1.98] |
| - Multivariable-adjusted Model 2 (95% CI) | 1 [ref.] | 1.26 [1.06-1.48] | 1.34 [1.11-1.63] | 0.80 [0.46-1.40] | 1.20 [0.80-1.81] |
| **Other supraventricular arrhythmia** | | | |  |  |
| N. of events | 401 | 89 | 60 | 12 | 17 |
| Incidence rate per 1000 PY (95% CI) | 0.51 [0.47-0.56] | 0.65 [0.53-0.79] | 0.59 [0.46-0.75] | 0.85 [0.49-1.40] | 0.79 [0.50-1.21] |
| Absolute rate difference, per 1000 PY (95% CI) | 0 [ref.] | 0.13 [-0.01-0.28] | 0.08 [-0.08-0.23] | 0.34 [-0.15-0.82] | 0.28 [-0.1-0.65] |
| - Minimally-adjusted Model 1 (95% CI) | 1 [ref.] | 1.33 [1.06-1.68] | 1.19 [0.90-1.58] | 2.52 [1.28-4.96] | 1.43 [0.84-2.43] |
| - Multivariable-adjusted Model 2 (95% CI) | 1 [ref.] | 1.27 [1.00-1.62] | 1.13 [0.84-1.51] | 2.28 [1.13-4.59] | 1.26 [0.68-2.32] |
| **Cardiac arrest and ventricular arrhythmia** | | | |  |  |
| N. of events | 753 | 208 | 147 | 24 | 37 |
| Incidence rate per 1000 PY (95% CI) | 0.96 [0.90-1.03] | 1.51 [1.32-1.72] | 1.44 [1.23-1.68] | 1.70 [1.15-2.45] | 1.71 [1.24-2.30] |
| Absolute rate difference, per 1000 PY (95% CI) | 0 [ref.] | 0.55 [0.33-0.77] | 0.48 [0.24-0.72] | 0.74 [0.06-1.43] | 0.75 [0.19-1.3] |
| - Minimally-adjusted Model 1 (95% CI) | 1 [ref.] | 1.84 [1.57-2.14] | 1.75 [1.46-2.10] | 2.34 [1.47-3.74] | 1.94 [1.34-2.80] |
| - Multivariable-adjusted Model 2 (95% CI) | 1 [ref.] | 1.53 [1.30-1.80] | 1.54 [1.27-1.86] | 1.71 [1.05-2.81] | 1.51 [0.98-2.31] |
|  |  |  |  |  |  |

Abbreviations: MASLD, metabolic dysfunction-associated steatotic liver disease; MASH, metabolic dysfunction-associated steatohepatitis; N., number; PY, person years; HR, hazard ratio; CI, confidence interval; ref., referent

*MASLD was defined by liver histology. For definitions of MASLD and arrhythmia outcomes, please see the Methods and the eMethods.

^1^ Confidence intervals for incidence rates and absolute rate differences were approximated by the normal distribution. Incidence rate difference is per 1000 person years.

^2^ The minimally-adjusted model 1 accounted for matching factors (age at the index date, sex, calendar year and county of residence). The fully-adjusted multivariable model accounted for the minimal model plus education, the number of recorded hospital visits in the 1 year prior to the index date (or corresponding matching date), and covariates defined up to and including the index date (i.e. diabetes, obesity, hypertension, dyslipidemia, chronic kidney disease and family history of cardiovascular disease before age 50 years), and alcohol use disorder during follow-up (defined as a time-varying covariate). For definitions, see the eMethods and Table S3.

**Table S6.** Incident Overall Arrhythmias and Atrial Fibrillation among MASLD Patients and Matched Full Sibling Comparators

| **Outcome** | | **Full Sibling Comparators**  **(N=9,837)** | | | **MASLD**  **(N=5,055)** |
| --- | --- | --- | --- | --- | --- |
| **Overall Arrhythmias** | | | | | |
| N. of events | | 760 | | | 494 |
| Incidence rate per 1000 PY (95% CI) | | 5.13 [4.78-5.50] | | | 7.47 [6.84-8.15] |
| Absolute rate difference, per 1000 PY (95% CI) | | 0 (ref.) | | | 2.34 [1.59-3.09] |
| Minimally-adjusted Model 1 (95% CI) | | 1 (ref.) | | | 1.55 [1.35-1.78] |
| Multivariable-adjusted Model 2 (95% CI) | | 1 (ref.) | | | 1.34 [1.15-1.56] |
| **Atrial Fibrillation** |  | | | | |
| N. of events | | 571 | | | 368 |
| Incidence rate per 1000 PY (95% CI) | | 3.83 [3.53-4.15] | | | 5.52 [4.98-6.10] |
| Absolute rate difference, per 1000 PY (95% CI) | | 0 (ref.) | | | 1.68 [1.04-2.33] |
| Minimally-adjusted Model 1 (95% CI) | | 1 (ref.) | | | 1.55 [1.32-1.82] |
| Multivariable-adjusted Model 2 (95% CI) | | 1 (ref.) | | | 1.32 [1.11-1.58] |
| **Bradyarrhythmias** | | | |  | |
| N. of events | | 100 | | | 63 |
| Incidence rate per 1000 PY (95% CI) | | 0.66 [0.54-0.80] | | | 0.92 [0.72-1.16] |
| Absolute rate difference, per 1000 PY (95% CI) | | 0 (ref.) | | | 0.26 [0-0.52] |
| Minimally-adjusted Model 1 (95% CI) | | 1 (ref.) | | | 1.45 [0.99-2.13] |
| Multivariable-adjusted Model 2 (95% CI) | | 1 (ref.) | | | 1.30 [0.84-2.03] |
| **Other Supraventricular Arrhythmias** | | |  | | |
| N. of events | | 89 | | | 38 |
| Incidence rate per 1000 PY (95% CI) | | 0.59 [0.48-0.72] | | | 0.56 [0.41-0.75] |
| Absolute rate difference, per 1000 PY (95% CI) | | 0 (ref.) | | | -0.03 [-0.25-0.18] |
| Minimally-adjusted Model 1 (95% CI) | | 1 (ref.) | | | 1.11 [0.73-1.70] |
| Multivariable-adjusted Model 2 (95% CI) | | 1 (ref.) | | | 1.11 [0.71-1.74] |
| **Ventricular arrhythmias/Cardiac Arrest** | | |  | | |
| N. of events | | 99 | | | 87 |
| Incidence rate per 1000 PY (95% CI) | | 0.65 [0.54-0.79] | | | 1.27 [1.03-1.55] |
| Absolute rate difference, per 1000 PY (95% CI) | | 0 (ref.) | | | 0.62 [0.32-0.92] |
| Minimally-adjusted Model 1 (95% CI) | | 1 (ref.) | | | 2.19 [1.55-3.09] |
| Multivariable-adjusted Model 2 (95% CI) | | 1 (ref.) | | | 1.89 [1.25-2.86] |
|  | |  | | |  |

Abbreviations: MASLD, metabolic dysfunction-associated steatotic liver disease; MASH, metabolic dysfunction-associated steatohepatitis; N., number; PY, person years; HR, hazard ratio; CI, confidence interval; ref., referent

*MASLD was defined by liver histology. For definitions of MASLD and arrhythmia outcomes, please see the Methods and the eMethods.

^1^ Confidence intervals for incidence rates and absolute rate differences were approximated by the normal distribution. Incidence rate difference is per 1000 person years.

^2^ The minimally-adjusted model 1 accounted for matching factors (age at the index date, sex, calendar year and county of residence). The fully-adjusted multivariable model accounted for the minimal model plus education, the number of recorded hospital visits in the 1 year prior to the index date (or corresponding matching date), and covariates defined up to and including the index date (i.e. diabetes, obesity, hypertension, dyslipidemia, chronic kidney disease and family history of cardiovascular disease before age 50 years), and alcohol use disorder during follow-up (defined as a time-varying covariate). For definitions, see the eMethods and Table S3.

**Table S7.** Incident Arrhythmia Outcomes among Adults with Histologically-Confirmed MASLD* and Matched Population Controls from 2006-2016 with Comprehensive Prescription Medication Use Data

| **Outcome** | **Population Controls**  **(n=9,757)** | **All MASLD**  **(n=2,375)** |
| --- | --- | --- |
| **Overall Arrhythmia** |  |  |
| N. of events | 395 | 117 |
| Incidence rate per 1000 PY (95% CI) | 7.44 [6.74-8.19] | 10.94 [9.13-13.01] |
| Absolute rate difference, per 1000 PY (95% CI) | 0 [ref.] | 3.5 [1.39-5.61] |
| Minimally-adjusted Model 1 (95% CI) | 1 [ref.] | 1.61 [1.31-1.98] |
| Multivariable-adjusted Model 1 (95% CI) | 1 [ref.] | 1.47 [1.20-1.81] |
|  |  |  |
| **Atrial Fibrillation** |  |  |
| N. of events | 313 | 84 |
| Incidence rate per 1000 PY (95% CI) | 5.87 [5.25-6.53] | 7.80 [6.30-9.55] |
| Absolute rate difference, per 1000 PY (95% CI) | 0 [ref.] | 1.93 [0.14-3.72] |
| Minimally-adjusted Model 1 (95% CI) | 1 [ref.] | 1.46 [1.15-1.86] |
| Multivariable-adjusted Model 1 (95% CI) | 1 [ref.] | 1.33 [1.05-1.70] |
|  |  |  |

Abbreviations: MASLD, metabolic dysfunction-associated steatotic liver disease; MASH, metabolic dysfunction-associated steatohepatitis; N., number; PY, person years; HR, hazard ratio; CI, confidence interval; ref., referent

*MASLD was defined by liver histology. For definitions of MASLD and arrhythmia outcomes, please see the Methods and the eMethods.

^1^ Confidence intervals for incidence rates and absolute rate differences were approximated by the normal distribution. Incidence rate difference is per 1000 person years.

^2^ The minimally-adjusted model 1 accounted for matching factors (age at the index date, sex, calendar year and county of residence). The fully-adjusted multivariable model accounted for the minimal model plus education, the number of recorded hospital visits in the 1 year prior to the index date (or corresponding matching date), and covariates defined up to and including the index date (i.e. diabetes, obesity, hypertension, dyslipidemia, chronic kidney disease and family history of cardiovascular disease before age 50 years), as well as use of aspirin, other anti-platelet agents, oral anticoagulants, statins, anti-hypertensive medications and/or anti-diabetic medications. For definitions, see the eMethods and Table S3.

**Table S8.** Incident Cardiac Arrhythmias among MASLD Patients and Matched Population Controls, after Excluding Patients with a Primary Outcome Within <90 Days*

|  | **Population Controls**  **(n=48,755)** | **All MASLD**  **(n=10,548)** | **Simple Steatosis**  **(n=7,162)** | **MASH without fibrosis**  **(n=1,190)** | **Non-Cirrhotic Fibrosis**  **(n=1,629)** | **Cirrhosis**  **(n=567)** |
| --- | --- | --- | --- | --- | --- | --- |
| **Overall Arrhythmia** | | | | |  |  |
| N. of events | 5,939 | 1,329 | 933 | 133 | 182 | 81 |
| Incidence rate per 1000 PY (95% CI) | 8.27 [8.06-8.48] | 10.14 [9.61-10.70] | 9.61 [9.01-10.24] | 9.84 [8.31-11.58] | 11.67 [10.10-13.43] | 16.79 [13.52-20.64] |
| Absolute rate difference, per 1000 PY (95% CI) | 0 (ref.) | 1.88 [1.29-2.46] | 1.34 [0.69-1.99] | 1.58 [-0.11-3.26] | 3.4 [1.7-5.11] | 8.52 [4.86-12.19] |
| Minimally-adjusted Model 1 (95% CI) | 1 (ref.) | 1.43 [1.35-1.52] | 1.38 [1.28-1.48] | 1.49 [1.23-1.81] | 1.47 [1.25-1.73] | 2.03 [1.59-2.59] |
| Multivariable-adjusted Model 2 (95% CI) | 1 (ref.) | 1.28 [1.20-1.36] | 1.25 [1.16-1.35] | 1.35 [1.11-1.65] | 1.25 [1.05-1.49] | 1.61 [1.21-2.15] |

Abbreviations: MASLD, metabolic dysfunction-associated steatotic liver disease; MASH, metabolic dysfunction-associated steatohepatitis; N., number; PY, person years; HR, hazard ratio; CI, confidence interval; ref., referent

*MASLD was defined by liver histology. For definitions of MASLD and arrhythmia outcomes, please see the Methods and the eMethods. For this analysis, follow-up began on day 90.

^1^ Confidence intervals for incidence rates and absolute rate differences were approximated by the normal distribution. Incidence rate difference is per 1000 person years.

^2^ The minimally-adjusted model 1 accounted for matching factors (age at the index date, sex, calendar year and county of residence). The fully-adjusted multivariable model accounted for the minimal model plus education, the number of recorded hospital visits in the 1 year prior to the index date (or corresponding matching date), and covariates defined up to and including the index date (i.e. diabetes, obesity, hypertension, dyslipidemia, chronic kidney disease and family history of cardiovascular disease before age 50 years), and alcohol use disorder during follow-up (defined as a time-varying covariate). For definitions, see the eMethods and Table S3.

**Table S9.** Incident Cardiac Arrhythmias among MASLD Patients and Matched Population Controls, after Excluding Patients with a Primary Outcome Within <2 years*

|  | **Population Comparators**  **(n=41,748)** | **All MASLD**  **(n=9,165)** | **Simple Steatosis**  **(n=6,297)** | **MASH without fibrosis**  **(n=1,033)** | **Non-Cirrhotic Fibrosis**  **(n=1,380)** | **Cirrhosis**  **(n=455)** |
| --- | --- | --- | --- | --- | --- | --- |
| **Overall Arrhythmia** | | | | |  |  |
| N. of events | 4,867 | 1,203 | 858 | 122 | 156 | 67 |
| Incidence rate per 1000 PY (95% CI) | 7.36 [7.16-7.57] | 9.28 [8.77-9.81] | 8.92 [8.34-9.52] | 9.14 [7.66-10.83] | 10.18 [8.70-11.84] | 14.22 [11.21-17.82] |
| Absolute rate difference, per 1000 PY (95% CI) | 0 (ref.) | 1.92 [1.36-2.48] | 1.55 [0.92-2.19] | 1.78 [0.14-3.41] | 2.82 [1.21-4.43] | 6.86 [3.45-10.27] |
| Minimally-adjusted Model 1 (95% CI) | 1 (ref.) | 1.41 [1.33-1.50] | 1.36 [1.26-1.46] | 1.57 [1.28-1.92] | 1.45 [1.21-1.72] | 1.95 [1.49-2.56] |
| Multivariable-adjusted Model 2 (95% CI) | 1 (ref.) | 1.27 [1.19-1.36] | 1.24 [1.15-1.35] | 1.45 [1.17-1.79] | 1.24 [1.02-1.49] | 1.57 [1.14-2.15] |

Abbreviations: MASLD, metabolic dysfunction-associated steatotic liver disease; MASH, metabolic dysfunction-associated steatohepatitis; N., number; PY, person years; HR, hazard ratio; CI, confidence interval; ref., referent

*MASLD was defined by liver histology. For definitions of MASLD and arrhythmia outcomes, please see the Methods and the eMethods. For this analysis, follow-up started at 2 years.

^1^ Confidence intervals for incidence rates and absolute rate differences were approximated by the normal distribution. Incidence rate difference is per 1000 person years.

^2^ The minimally-adjusted model 1 accounted for matching factors (age at the index date, sex, calendar year and county of residence). The fully-adjusted multivariable model accounted for the minimal model plus education, the number of recorded hospital visits in the 1 year prior to the index date (or corresponding matching date), and covariates defined up to and including the index date (i.e. diabetes, obesity, hypertension, dyslipidemia, chronic kidney disease and family history of cardiovascular disease before age 50 years), and alcohol use disorder during follow-up (defined as a time-varying covariate). For definitions, see the eMethods and Table S3.

**Table S10.** Incident Primary Cardiac Arrhythmias among MASLD Patients and Matched Population Controls, after Excluding Patients with Underlying CVD and Censoring at the Date of CVD Diagnoses in Follow-up*

|  | **Population Comparators**  **(n=44,666)** | **All MASLD**  **(n=10,104)** | **Simple Steatosis**  **(n=6,937)** | **MASH without fibrosis**  **(n=1,140)** | **Non-Cirrhotic Fibrosis**  **(n=1,503)** | **Cirrhosis**  **(n=524)** |
| --- | --- | --- | --- | --- | --- | --- |
| **Overall Arrhythmia** | | | | |  |  |
| N. of events | 3698 | 789 | 559 | 77 | 101 | 52 |
| Incidence rate per 1000 PY (95% CI) | 5.85 [5.66-6.04] | 7.04 [6.57-7.54] | 6.69 [6.16-7.25] | 6.60 [5.28-8.15] | 7.84 [6.46-9.44] | 13.48 [10.29-17.39] |
| Absolute rate difference, per 1000 PY (95% CI) | 0 (ref.) | 1.20 [0.67-1.72] | 0.84 [0.25-1.42] | 0.75 [-0.74-2.24] | 1.99 [0.45-3.53] | 7.64 [3.97-11.31] |
| Minimally-adjusted Model 1 (95% CI) | 1 (ref.) | 1.42 [1.31-1.53] | 1.37 [1.25-1.50] | 1.40 [1.10-1.80] | 1.38 [1.11-1.72] | 2.38 [1.75-3.25] |
| Multivariable-adjusted Model 2 (95% CI) | 1 (ref.) | 1.29 [1.19-1.40] | 1.29 [1.17-1.42] | 1.30 [0.99-1.71] | 1.13 [0.89-1.43] | 2.00 [1.40-2.86] |

Abbreviations: MASLD, metabolic dysfunction-associated steatotic liver disease; MASH, metabolic dysfunction-associated steatohepatitis; N., number; PY, person years; HR, hazard ratio; CI, confidence interval; ref., referent

*MASLD was defined by liver histology. For definitions of MASLD and arrhythmia outcomes, please see the Methods and the eMethods. For this analysis, any person with cardiovascular disease recorded on or prior to the index/matching date was excluded; further, any person with incident CVD recorded during follow-up was censored at the CVD diagnosis date. For details, see Methods.

^1^ Confidence intervals for incidence rates and absolute rate differences were approximated by the normal distribution. Incidence rate difference is per 1000 person years.

^2^ The minimally-adjusted model 1 accounted for matching factors (age at the index date, sex, calendar year and county of residence). The fully-adjusted multivariable model accounted for the minimal model plus education, the number of recorded hospital visits in the 1 year prior to the index date (or corresponding matching date), and covariates defined up to and including the index date (i.e. diabetes, obesity, hypertension, dyslipidemia, chronic kidney disease and family history of cardiovascular disease before age 50 years), and alcohol use disorder during follow-up (defined as a time-varying covariate). For definitions, see the eMethods and Table S3.

**Supplementary Figure:**

**Figure S1.** Construction of the Nationwide Matched Cohort


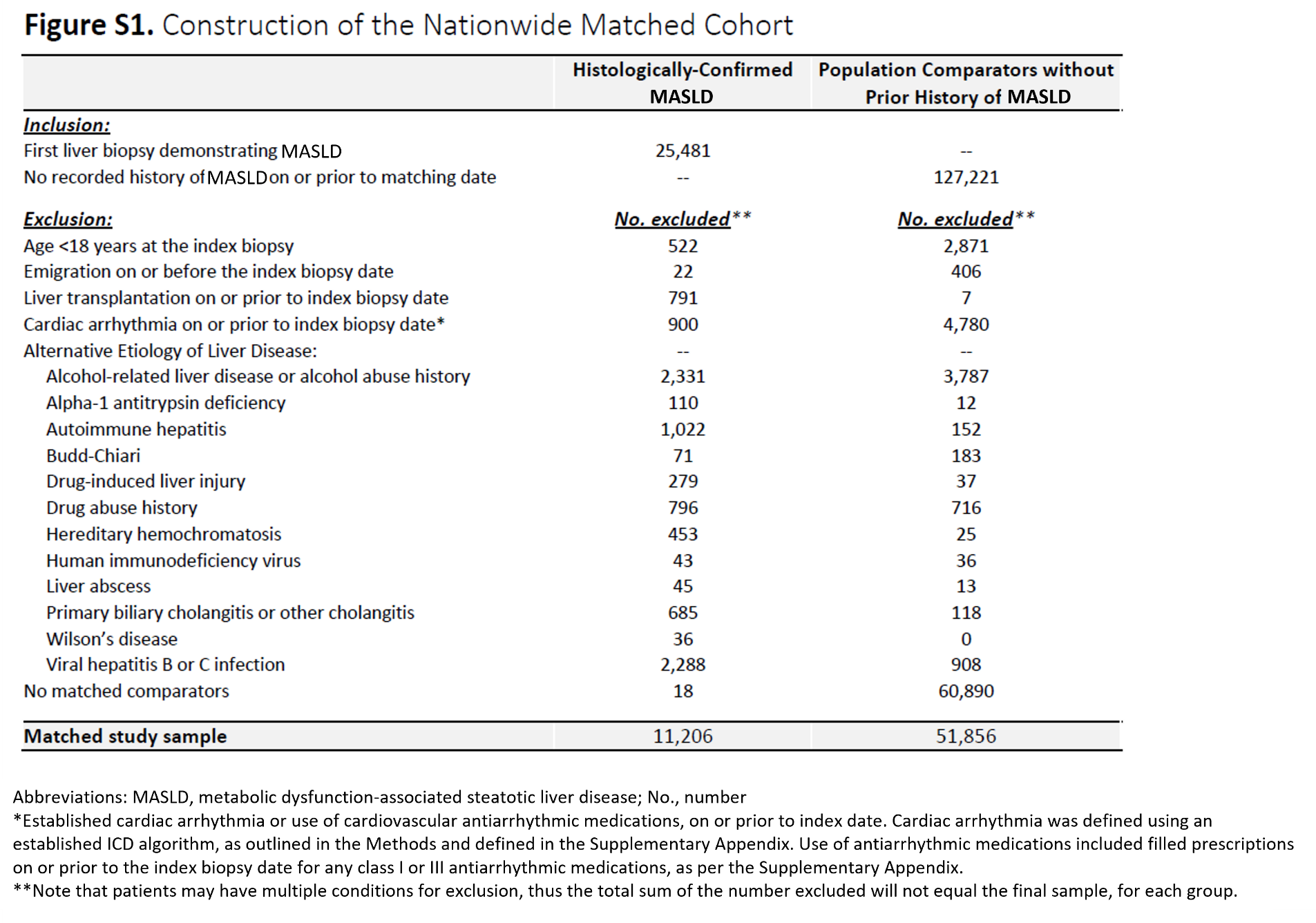


**Supplementary References:**

1. Svensk Förening för Patologi – Svensk Förening för Klinisk Cytologi V, April 4, 2019. <http://www.svfp.se/foreningar/uploads/L15178/kvast/lever/Leverbiopsier2019.pdf>; Accessed November 1, 2019. .

2. Simon TG, Roelstraete B, Khalili H, Hagstrom H, Ludvigsson JF. Mortality in biopsy-confirmed nonalcoholic fatty liver disease: results from a nationwide cohort. *Gut*. Oct 9 2020;doi:10.1136/gutjnl-2020-322786

3. Ludvigsson JF, Svedberg P, Olen O, Bruze G, Neovius M. The longitudinal integrated database for health insurance and labour market studies (LISA) and its use in medical research. *Eur J Epidemiol*. Apr 2019;34(4):423-437. doi:10.1007/s10654-019-00511-8

4. Simon TG, Duberg AS, Aleman S, et al. Lipophilic Statins and Risk for Hepatocellular Carcinoma and Death in Patients With Chronic Viral Hepatitis: Results From a Nationwide Swedish Population. *Ann Intern Med*. Aug 20 2019;doi:10.7326/M18-2753
